# Ex vivo factor VIII‐modified proliferating human hepatocytes therapy for haemophilia A

**DOI:** 10.1111/cpr.13467

**Published:** 2023-05-17

**Authors:** Kun Zhang, Ning Wu, Jing Cen, Jie Li, Zhen Wang, Qiang Xia, Lijian Hui

**Affiliations:** ^1^ State Key Laboratory of Cell Biology, CAS Center for Excellence in Molecular Cell Science, Shanghai Institute of Biochemistry and Cell Biology, Chinese Academy of Sciences University of Chinese Academy of Sciences Shanghai China; ^2^ Department of Liver Surgery, Renji Hospital, School of Medicine Shanghai Jiao Tong University Shanghai China; ^3^ Hangzhou Institute for Advanced Study University of Chinese Academy of Sciences Hangzhou China; ^4^ Institute for Stem Cell and Regeneration Chinese Academy of Sciences Beijing China; ^5^ School of Life Science and Technology ShanghaiTech University Shanghai China

## Abstract

Ex vivo gene manipulation in human hepatocytes is a promising therapeutic strategy in the treatment of inherited liver diseases. However, a major limitation is the lack of a highly efficient and safe genetic manipulation system for transplantable primary human hepatocytes (PHHs). Here, we reported that proliferating human hepatocytes (ProliHHs) cultured in vitro showed high susceptibility to lentivirus‐mediated genetic modification and maintained cellular phenotypes after lentiviral infection. Human factor VIII expression was introduced through F8‐Lentivirus‐mediated transduction of ProliHHs followed by xenotransplantation into immunocompromised haemophilia A mice. We demonstrated that these F8‐modified ProliHHs could effectively repopulate the mouse liver, resulting in therapeutic benefits in mouse models. Furthermore, no genotoxicity was detected in F8‐modified ProliHHs using lentiviral integration site analysis. Thus, this study demonstrated, for the first time, the feasibility and safety of lentiviral modification in ProliHHs to induce the expression of coagulation factor VIII in the treatment of haemophilia A.

## Graphical abstract

Hui et al. found that Proliferating Human Hepatocytes (ProliHHs) cultured in vitro showed high susceptibility to lentivirus‐mediated genetic manipulation, and F8‐modified ProliHHs could effectively repopulate the liver and alleviate symptoms of mouse models with haemophilia A without detecting genotoxicity. This study provides evidence for the potential treatment of monogenic liver diseases using gene‐modified ProliHHs therapy.

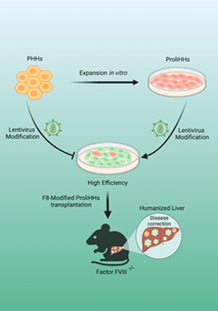

## 1 INTRODUCTION

Haemophilia A is a severe X‐linked recessive inherited bleeding disorder caused by a deficiency in coagulation factor VIII (FVIII), a critical protein secreted by liver endothelial cells that is involved in the blood coagulation cascade.^1^ Haemophilia A is a prevalent bleeding disorder (1:5000 male births) and represents 80%–85% of the entire haemophilia population.^2^ Based on residual factor activity, haemophilia A can be classified as severe (<1% normal FVIII activity), moderate (1%–5%), or mild (5%–50%).^3^ Patients usually experience spontaneous bleeding into joints and soft tissue, eventually resulting in disabling hemophilic arthropathy and early death, especially in cases of severe haemophilia. Currently, the mainstay of haemophilia A therapy is protein replacement of deficient FVIII using either conventional or extended half‐life FVIII to achieve sufficient levels for prophylaxis or on‐demand therapy in response to bleeding.^4^ Despite significant progress, replacement therapy requires repeated administration and may induce severe adverse events, such as blood virus infections (HBV, HCV and HIV) and the generation of neutralizing antibodies against FVIII.^5^ Therefore, further improvements are warranted in the treatment of patients with haemophilia A.

Haemophilia A is an ideal target disease for gene or cell therapy, given that the quality of life can be significantly improved in patients with circulating FVIII activity >5%. Furthermore, gene therapy provides an alternative approach—eliminating the disadvantages of protein replacement—and has the potential of being a one‐time, life‐long, disease‐altering therapy. In the last four decades, numerous efforts and remarkable advances have been made in the treatment of haemophilia A. Previous studies have shown that Adeno‐associated virus (AAV) vectors injected into the liver or muscle can produce FVIII at a therapeutic level and ameliorate disease symptoms in mouse and dog animal models of haemophilia A.^6^, ^7^ Encouraged by these findings, researchers designing clinical trials have focused on in vivo clotting factor gene transfer to cure haemophilia using AAV delivery. According to recent clinical experiments, a wide range of FVIII activity (12%–250%) was observed in seven patients treated with AAV5‐FVIII.^8^, ^9^ However, the levels of F8 gene expression in treated patients were found to gradually decrease over 4 years (2016–2020), and whether these levels are in the final steady state remains to be determined. In addition, AAV‐mediated gene therapy is still hindered by problems; in particular, some patients can possess pre‐existing antibodies to AAV and the lack of clarity on the effects in paediatric patients with haemophilia remain unclear. Therefore, other research groups are investigating gene‐modified cell therapy approaches, which could avoid the critical obstacles of AAV therapy in vivo. F8‐modified haematopoietic stem cells and FVIII‐corrected endothelial cells derived from haemophilia A patient‐induced pluripotent stem cells also provide long‐term phenotypic correction of haemophilia A.^10^, ^11^, ^12^ These results suggest that ex vivo gene‐modified cell therapy is a promising strategy for haemophilia A treatment.

Hepatocytes can engraft and repopulate the liver post‐transplantation. Therefore, hepatocyte transplantation is a promising alternative to liver transplantation in the treatment of various genetic liver diseases.^13^, ^14^ Although liver sinusoidal endothelial cells, but not hepatocytes, are the primary cells of FVIII secretion,^15^, ^16^, ^17^ hepatocytes can still be considered as an ideal target cell for F8 expression, as they are the predominant host cells for gene therapy (AAV5‐hFVIII derived factor VIII protein expression).^9^ However, little progress has been made in gene‐modified hepatocyte therapy owing to the complicated and inefficient process of gene manipulation in primary human hepatocytes (PHHs).^18^

In this study, we examined the potential of F8‐modified human hepatocytes to treat haemophilia A in a mouse model. The results showed that proliferating human hepatocytes (ProliHHs) were more susceptible to lentivirus infection than PHHs. F8‐modified ProliHHs effectively secreted FVIII protein and maintained a cell state similar to that of untreated ProliHHs. Importantly, we demonstrated that F8‐modified ProliHHs transplantation could restore coagulation function to wild‐type (WT) mice, with implications for effective treatment. Furthermore, analysis of the integration profile indicated no genotoxicity with these lentiviral vectors (LVs). Thus, these findings provide proof of concept for the potential use of F8‐modified human hepatocytes in the treatment of haemophilia A.

## 2 MATERIALS AND METHODS

### 2.1 Human hepatocyte culture

We used cryopreserved human hepatocytes (Lot: MRW, JFC) from two individuals provided by Celsis In Vitro Technologies (Blatimore, MD). The culture protocol was adapted from our previously reported system with modifications.^19^ The cryopreserved PHHs were thawed in the 37°C‐water bath in Dulbecco's Modified Eagle Medium with 10% fetal bovine serum. The thawed hepatocytes were resuscitated and cultured in 37°C, Hypoxia incubator (5% CO_2_, 1% O_2_). The medium was changed to human HM about 24 h after seeding and every 2 days thereafter.

### 2.2 Lentivirus transfections

Modified lentivirus plasmids carrying Empty‐GFP (EV) or F8 were introduced into 293FT cells together with the third generation lentiviral packaging plasmid pRSV‐Rev (Addgene#12253) pMDLg/pRRE (Addgene#12251) and envelop plasmid pMD2.G (Addgene#12259) to produce viruses. The codon‐optimized DNA encoding a BDD human F8 was cloned into the pHIV‐EGFP vectors with EF1α promoter and GFP. Viruses (multiplicity of infection of 0, 1, 2, 4) were added into the medium of ProliHHs with the 5 μg/mL Polybrene.

### 2.3 Mice

The *Fah* ^*−/−*^ *Rag2* ^*−/−*^ *Il2rg* ^*−/−*^(FRG) mice were presented from Dr. Wang Xin's Lab. FRG mice are on the hybrid strain of C57BL/6J and 129S6/ SvEvTac. The mice were fed with drinking water containing 7.5 mg/L NTBC (Synthesized by Capot Chemical, China). A 8 to 12 weeks old male and female mice were used for experiments and we observe no sex bias differences were detected. *FVIII* ^*−/−*^ mice were purchased from Shanghai Biomodel Organism Center, Inc. A quadruple KO *Fah* ^*−/−*^ *Rag2* ^*−/−*^ *IL2rg* ^*−/−*^ *FVIII* ^*−/−*^ mouse line (FRGF8) was crossed by FRG mice and FVIII deficient haemophilia A mice.

None of the animals used in our study had been subjected to prior procedures and was drug and test naive. All animals were housed in a temperature‐ and light‐controlled (12‐h light/dark cycle) specific pathogen‐free animal facility, in individually ventilated cages always with companion mice. All animal experiments were performed basis on protocols approved by the institutional animal care and use committee at the Shanghai Institute of Biochemistry and Cell Biology.

### 2.4 Polymerase Chain Reaction (PCR)

Total RNA was isolated from cells and mice liver by Trizol (Invitrogen). RNA extracted from freshly thawed PHHs or untreated ProliHHs was used as standard controls. 500 ng RNA was reverse transcribed into cDNA with M‐MLV Reverse Transcriptase (Promega) according to recommended manufacturer's instructions. Genomic PCR was performed with Taq polymerase (TransGen). Quantitative real‐time PCR (Q‐PCR) was performed with SYBR Premix Ex Taq (TaKaRa) on ABI StepOnePlus real‐time PCR instrument (Applied Biosystems). All q‐PCR data were performed with at least three repeats. Primer sequences are listed in Table S1.

### 2.5 Human Albumin ELISA

To identify the protein secretion of human Albumin in transplantation experiments, transplanted FRGF8 animal serum was collected at multiple times. Protein levels of human albumin were measured by the human‐specific albumin ELISA Quantitation Set (Bethyl Laboratory) according to the manufacturer's instructions recommended by the Bethyl Laboratory. Mice serum was diluted in a range from 10‐ to 100,000‐fold to get actual values according to the linear range of the standard curve.

### 2.6 Human FVIII antigen and activity

Transplanted FRGF8 animal plasma was collected at multiple times using sodium citrate anticoagulant. Plasma human FVIII levels in mice were quantified by a VisuLize™ Factor VIII Antigen Kit (FVIII‐AG; Affinity Biologicals). Human FVIII levels are shown as a percentage of the normal level according to the manufacturer's protocol. FVIII activity was measured by an aPTT assay using a coagulation instrument (BJ MDC, MC‐4000) based on the manufacturer's protocol. Thirty microliter of mice plasma sample was mixed with 30 microliter of aPTT reagent (BJ MDC, 03209MC) followed by a 3‐min incubation at 37°C. The reaction was initiated after the adding 30 microliter of 25 mM calcium chloride (BJ MDC, 03311). The coagulation instrument recorded the time of clot formation. Normal mice plasma was used as a calibration sample.

### 2.7 IF staining

FRGF8 mice livers were fixed overnight in 4% neutral buffered paraformaldehyde (Solarbio), and then livers were embedded in paraffin. Five micrometre thick sections obtained from paraffin tissues were placed on adhesion microscope slides. Tissue sections were subjected to IF staining. We used a Tyramide signal amplification (TSA) fluorescein evaluation kit (Akoya) following standard protocols for the co‐immunostaining of tissue samples. For immunohistochemistry, deparaffinized and rehydrated slides were subjected to autoclave antigen retrieval in a 10 mmol/L citric acid buffer (pH 6.0) and cooled to room temperature. Slides were blocked with 3% H_2_O_2_ for 30 min, washed in phosphate‐buffered saline, then blocked with 5% normal goat serum in PBS. Slides were incubated with diluted primary antibodies overnight at 4°C.

For immunofluorescent staining, the lentivirus‐modified ProliHHs were fixed with 4% paraformaldehyde for 30 min at room temperature and then penetrated with PBS containing 0.2% Triton X‐100 (Sigma) for 15 min. ProliHHs were then washed three times with PBS. After being blocked by 3% BSA in PBS for 60 min at room temperature, ProliHHs were incubated with primary antibodies at 4°C overnight, washed three times with PBS, and then incubated with appropriate fluorescence‐conjugated secondary antibody for 60 min at room temperature in the dark. Nuclei were stained with DAPI (Sigma). Primary and secondary antibodies were diluted in PBS containing 3% BSA.

Antibodies used for immunofluorescent staining are as follows: rabbit anti‐human GAPDH (Abcam, ab128915, 1:1000), goat anti‐human‐Albumin (Bethyl Laboratories, 1:200), mouse anti‐Hnf4α (Santa Cruz, 1:200), rabbit anti‐FAH (gift from Dr.Wang xin's Lab, 1:3000), mouse anti‐human CYP3A4 (Santa Cruz, 1:200), mouse anti‐GS (BD, 1:100), rabbit anti‐SOX9 (Millipore, 1:1000), mouse anti‐CK19 (Genetex, 1:200), Sheep anti‐FVIII (Affinity Biologicals, 1:500), Cy5‐conjugated donkey anti‐goat IgG (Jackson Lab, 1:500), FITC‐conjugated donkey anti‐rabbit IgG (Jackson Lab, 1:500), Cy3‐conjugated donkey anti‐rabbit IgG (Jackson Lab, 1:500), Cy3‐conjugated donkey anti‐mouse IgG (Jackson Lab, 1:1000), FITC‐conjugated donkey anti‐mouse IgG (Jackson Lab, 1:1000).

### 2.8 Transplantation of cells into *Fah* ^−/*−*^ *Rag2* ^−/*−*^ *Il2rg* ^−/−^ FVIII ^−/−^mice

Before ProliHHs transplantation, the concentration of NTBC in drinking water for FRGF8 mice was totally withdrawn for another 6 days. Mice were administered 10 mg/kg Dex (Selleck, S1322, dissolved in 3% DMSO, 45% PEG‐300 and 52% saline) by intraperitoneal injection 5 min before transplantation on the first day and for the next.^20^ 6 × 10^5^ lentivirus‐mediated ProliHHs at passages (P4 and P5) in 150 microliter PBS were intrasplenically transplanted into FRGF8 mice. After transplantation, NTBC was transiently put on for 4 days when mice lost 10%–20% of their body weights. Mice were sacrificed at 5 months after transplantation.

### 2.9 LTA‐PCR and second‐generation sequencing system

LTM‐PCR was used to identify LV flanking genomic sequences. Illumina barcoded libraries were obtained from one independent pre‐graft F8‐modifed ProliHHs and two post‐F8‐modifed ProliHHs transplanted livers (humanized liver 1 and humanized liver 2). First, we used the ultrasonic DNA breaker (Covaris M220) to cut 1.5 μg each sample genomic DNA to an average length of 500 ± 50 bp (Tapestation 4150 is used for the quality control of DNA fragment). Fragmented DNA was recovered through purification and then the fragmented DNA was extended by the long terminal repeat (LTR) specific biotinylated primers. For linear PCR, one biotinylated primer [LV‐543‐bio‐Primer‐Sequence (5′‐3′) AGTGCTTCAAGTAGTGTGTGCC] was used to bind the 3′‐LTR regions of the viral genome. According to the availability of template DNA, a total of 1500 ng was used for the 3‐fold analysis per sample.

Subsequent steps involved Ampure XP magnetic capture of the biotinylated PCR products.

Fifty microlitre of streptavidin‐coated beads were administered to each PCR product and incubated overnight at 300 rpm on a shaker for 1 h. After incubation, the DNA‐bead complexes were ligated the known oligonucleotide sequence to an unknown DNA‐bead complex sequence. These samples were amplified by the second index PCR [SK‐LTR‐4‐bio‐Primer (5′‐3′) AGTAAGATGCCCGTGAGT and LC I‐Primer (5′‐3′) GACCCTGVAGATCTGAGATC].

After the first exponential PCR was finished, PCR products were captured by streptavidin‐coated beads. As with the first exponential PCR, 2 μL of the previous product was mixed with second exponential PCR primer [MiS‐LK1‐Primer (5′‐3′) AAGCAGAAGACGGCATACGAGATCGGTCTCGGCATTCCTGCCGGTCACGCTCTTCCGATCGTACGGCACAGCAGTTAGG and MiS3nrLV‐Primer (5′‐3′) AATGATACGGCGACCACCGAGATCTACACTCTTTCCTGACACGCGTATCTTCCGATCTGATGACTCAGAGACTTTTAGTC]. As final step, 50 ng of each sample was pooled. After measuring the concentration by Qubit, the average fragment size was measured with High Sensitivity D1000 Screen Tape Kit TapeStation. The pool was adjusted to 10 nM with EB buffer. The samples deal with LTA‐PCR were sequenced at Shanghai Waker Bioscience Co., Ltd. After obtained the raw sequencing data, which need first analysis with Genome Integration Site Analysis Pipeline.

Integration site sequence data were controlled for IS that were aligned to more than one definite location (multiple hits) and collision reads (identical IS found in different patients). The tool ISOT 1.0 (in‐house Waker Bioscience) generated a list of all unique IS with precise loci, gene names and sequence counts.

### 2.10 Evaluation of integrated cell cloning

Illumina MiSeq second generation sequencing platform could semi‐quantitatively evaluate the integrated cell cloning by determining the number of sequences (search frequency) of single vector‐genome integration sites. The relative sequence count of all detected ISs is calculated based on all sequences that can be mapped to specific locations in the genome. For each sample, analyse the top 10 most significant ISs (ranking from 1 to 10, from high to low).

### 2.11 Analysis of clone diversity

We developed a method based on the property of Rényi entropy to evaluate the diversity of each sample cell clone. The algorithm calculates α Take the H value under two extremes: richness and evenness, and construct a cloning plane. The distance ratio between theoretical maximum polyclonal degree and monoclonal degree defines the polyclonal monoclonal distance, which is a diversity measurement developed internally and can accurately evaluate the sample richness and uniformity.

### 2.12 Relationship between integration sites and tumour related genes

We compiled a well‐defined list of cancer genes from the cancer census database (http://cancer.sanger.ac.uk/census). The selection of these genes is based on how mutations in these genes promote cancer progression: (1) amplification, (2) large deletions or (3) splice site mutations. In addition, genes identified as partners of carcinogenic gene translocation were also selected. Obtain cancer gene data from Ensembl human genes (chromosome, gene start, gene end, chain, gene name, transcript count, transcription start site [TSS] and transcript support level [TSL]) (http://www.ensembl.org/biomart/martview/; GRCh38 version.p10). Only TSS from transcripts, where all splicing connections are made by at least one non suspect mRNA (TSL1; https://www.ensembl.org/Help/Glossary?id=492). The genes detected in the 100 kb window of the TSS of the pre‐selected cancer related genes and SAE genes are mapped by the occurrence frequency in the total reading amount of the sample control.

### 2.13 Statistical analyses

The number of biological and technical replicates and animals are indicated in figure legends and text. All data are presented as the mean ± SD. For most statistic evaluation, an unpaired Student's *t* test was applied for calculating statistical probability in this study. *p* Values were calculated by two‐tailed test. Only for survival analysis in Figure 5B,G, the Mantel‐Cox log‐rank test was applied. Statistic calculation was performed using GraphPad Prism9 (GraphPad).

## 3 RESULTS

### 3.1 ProliHHs are highly susceptible to lentiviral infection

Gene manipulation of PHHs is challenging and inefficient, which greatly limits the potential application of gene‐modified human hepatocytes for cell therapy and in vitro modelling of liver diseases.^18^, ^21^, ^22^ Recently, we established a hepatocyte medium (HM) culture system that allowed 10,000‐fold expansion of PHHs. These ProliHHs have a bi‐phenotypic ‘intermediate’ status between mature hepatocytes and liver progenitor cells.^19^ Therefore, we speculate that ProliHHs may be more amenable to gene manipulation for various applications.

**FIGURE 1:**
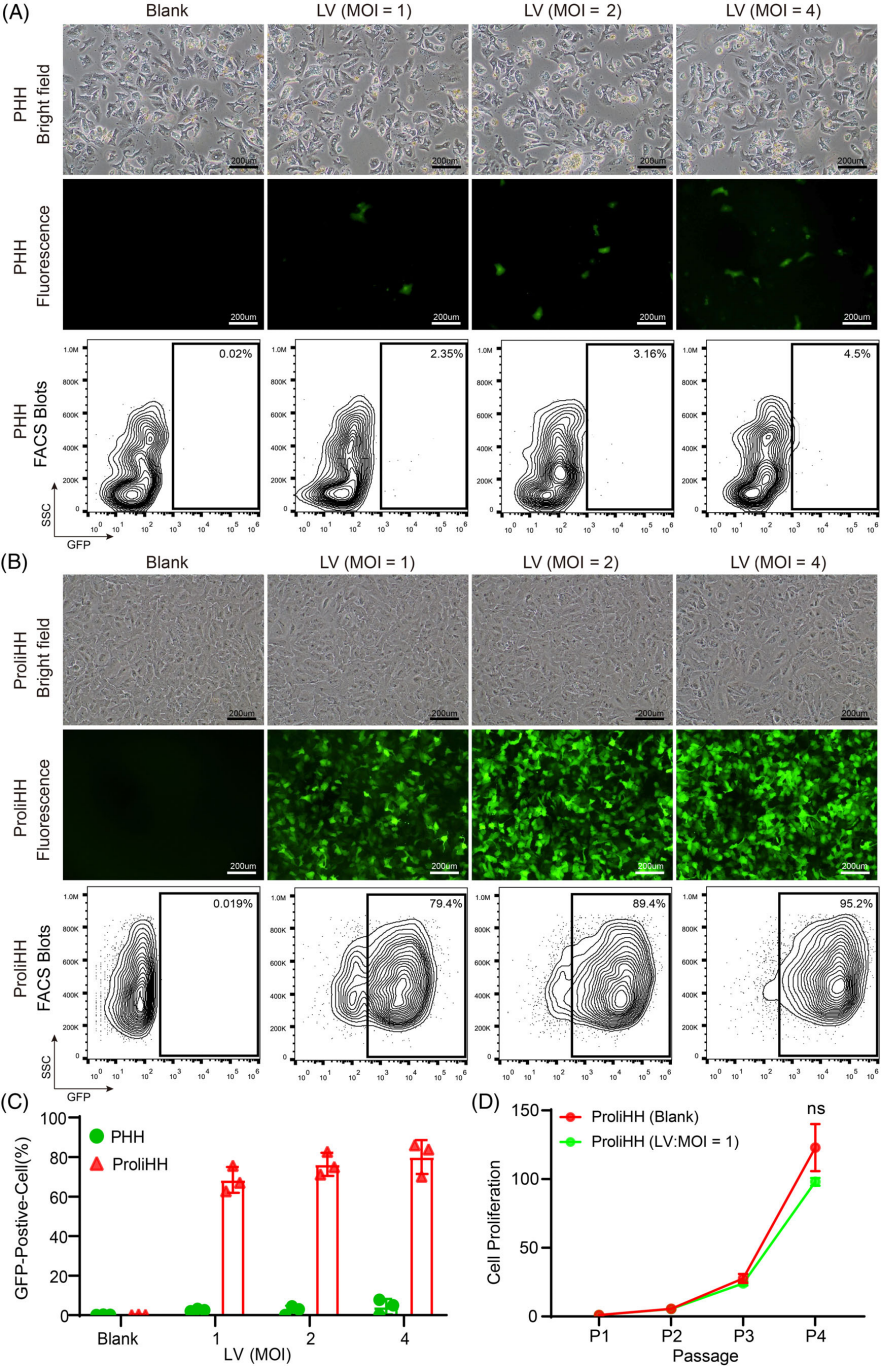
Proliferating human hepatocytes (ProliHHs) are highly susceptible to lentiviral infection. (A) Representative images of primary human hepatocytes (Lot: MRW) in brightfield and fluorescence microscope. Hepatocytes were infected with Empty‐lentivirus (LV) expressing green fluorescent protein (GFP) at multiplicities of infections (MOIs) of 0, 1, 2 and 4 for 3 days in Dulbecco's Modified Eagle Medium. Scale bars, 200 μm. Flow cytometry was performed 48 h after LV transduction. (B) Representative images of ProliHHs (lot: JFC) in brightfield and fluorescence microscope. ProliHHs were infected with Empty‐LV expressing GFP at MOIs of 0, 1, 2 and 4 for 3 days in hepatocyte medium (HM). Scale bars, 200 μm. Flow cytometry was performed 48 h after LV transduction. (C) Percentage of GFP‐positive cells under different titers of Empty‐LV expressing GFP were analysed by the flow cytometry. Data are means ± SD (*n* = 3 replicates). (D) Growth curves of Empty‐LV‐infected ProliHHs (MOI = 1) were analysed at indicated passages in HM. ProliHHs without LV infection were used as a control. The data are shown as the mean ± SD. NS > 0.05, Student's *t* test.

Lentiviruses are generally the most convenient and commonly used tools for genetic manipulation in the basic research and clinical treatment of life science, such as gene overexpression, knockdown, and cell marking.^23^ Here, we used PHHs as a negative control and tested the susceptibility of ProliHHs to lentiviral infection. PHHs and ProliHHs were infected with lentiviruses encoding green fluorescent protein (GFP) at different multiplicities of infection (MOI). Surprisingly, ProliHHs were highly susceptible to lentiviral infection. Even when infected with a low viral titre (MOI = 1), the majority of ProliHHs were found to express GFP, as was evidenced by cell fluorescence and flow cytometry. In addition, the higher viral titre lentivirus (MOI = 4) further enhanced the infection rate of ProliHHs, with its infection efficiency close to 100% (Figure 1B). By contrast, under a high viral titre (MOI = 4), PHHs showed a typically low efficiency rate (<5%) in terms of lentiviral infection (Figure 1A). These results strongly indicated that ProliHHs were more susceptible to lentivirus infection than PHHs. Furthermore, the ProliHHs from multiple individuals had comparable lentivirus infection rates, which indicated that lentivirus infection of ProliHHs was donor‐independent (Figure 1C). This susceptibility phenotype may be related to the strong proliferation ability and bi‐phenotypic ‘intermediate’ status of ProliHHs. Generally, infection with a high‐titre lentivirus could damage cells or even cause cell death. Therefore, it is critical to maintain cell characteristics after lentivirus modification. For this purpose, ProliHHs were subjected to lentivirus infection (MOI = 1) and the expansion of lentivirus infected ProliHHs was assessed during passages. The statistical results showed no significant difference in cell proliferation between the infected and uninfected ProliHHs (Figure 1D). However, we also observed that high‐titre lentivirus infection (MOI = 4) significantly hindered cell proliferation and induced the formation of more giant cells (Figure 1B). With these findings, we showed that ProliHHs were prone to infection by lentivirus, and the low titre lentivirus could maintain the original characteristics of ProliHHs after achieving efficient gene manipulation.

### 3.2 F8‐lentiviral modification maintains features of ProliHHs

**FIGURE 2:**
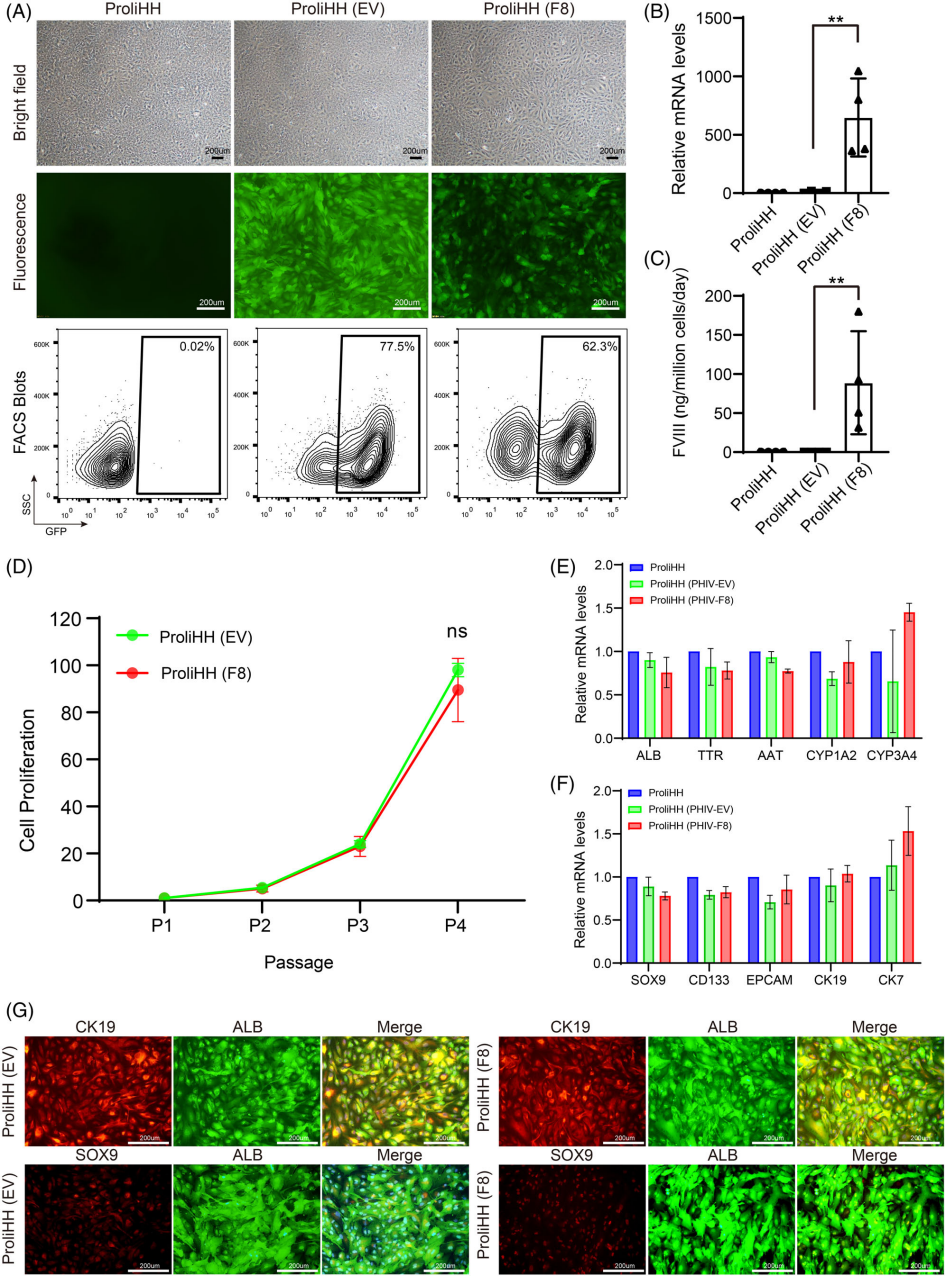
F8‐lentiviral modification maintain features of proliferating human hepatocytes (ProliHHs). (A) Representative images of untreated ProliHHs, Empty‐lentivirus (EV)‐infected ProliHHs and F8‐lentivirus (F8)‐infected ProliHHs (lot: JFC‐Passage4) in brightfield and fluorescence microscope. ProliHHs were infected with lentivirus (LV) (MOI = 1) for 5 days in hepatocyte medium (HM). Scale bars, 200 μm. Flow cytometry was performed 5 days after LV transduction. (B) The expression of human F8 in ProliHHs after infecting F8‐Lentivirus was determined by qPCR. Data are normalized to untreated ProliHHs. The data are shown as the mean ± SD. **p < 0.01, Student's *t* test. (C) The human FVIII secretion of ProliHHs after infecting F8‐Lentivirus was measured by human FVIII ELISA. The data are shown as the mean ± SD. **p < 0.01, Student's *t* test. (D) Growth curves of EV/F8‐infected ProliHHs (MOI = 1) were analysed at indicated passages in HM. ProliHHs infected EV was used as a control. The data are shown as the mean ± SD. NS > 0.05, Student's *t* test. (E) Hepatic gene expressions of ProliHHs after EV/F8‐lentivirus infection was measured by qPCR. Data are normalized to untreated ProliHHs. (F) Progenitor associated markers of ProliHHs after EV/F8‐lentivirus infection was measured by qPCR. Data are normalized to untreated ProliHHs. (G) The co‐expression of the mature hepatic marker ALB and progenitor‐associated markers such as SOX9 and CK19 was determined by immunofluorescent staining in ProliHHs infected by LV at P4. Scale bars, 200 μm.

To test the hypothesis that F8‐modified ProliHHs have therapeutic potential for the treatment of haemophilia A, we first confirmed that PHHs and ProliHHs do not express F8 through analysis of cellular transcriptional profiling (Figure S1A), which was a prerequisite for the F8‐modified ProliHH therapy. Next, we constructed a new third‐generation self‐inactivating LV carrying an EF1α promoter, a codon‐optimized DNA encoding a B domain‐deleted (BDD) human FVIII protein, and a reporter GFP to indicate the infection efficiency (Figure S1B). The packaging capacity of LVs readily accommodated these expression cassettes, and high‐titre F8‐lentiviral particles were produced using a lentiviral packaging system. Using untreated cells as a negative control, we found that the efficiency of F8‐lentivirus‐infected ProliHHs (F8‐ProliHHs) was approximately 65%, which was lower than that of empty lentivirus‐infected ProliHHs (EV‐ProliHHs), likely due to the large size of the F8 gene (4375 bp) (Figure 2A). To investigate whether F8‐ProliHH was functional, F8 gene expression and protein secretion were separately tested in F8‐modified ProliHHs. We found that F8 showed a significant increase as determined by F8‐qPCR and ELISA (Figure 2B,C). In addition, we analysed the characteristics of ProliHHs before and after F8 modification, including the mRNA expression and protein immunofluorescent staining of hepatic‐function‐ and progenitor‐associated signature genes (Figure 2E,G) and found that these characteristics were not affected by F8 modification. Together, these findings suggested that F8‐ProliHHs gained the ability to secrete FVIII proteins, and F8 modification did not affect the bi‐phenotypic status of ProliHHs.

### 3.3 Transplantation of F8‐modified ProliHHs results in functional rescue in haemophilia A mice

We examined whether F8‐ProliHHs were viable and functional in the treatment of a mouse model of haemophilia A. For this purpose, we generated a quadruple knockout mouse model of haemophilia A that allowed the engraftment and expansion of human hepatocytes. As reported, *Fah* ^−/−^ *Rag2* ^−/−^ *IL2rg* ^−/−^ (FRG) mice are classical immune‐deficient models that characterize the engraftment and repopulation of transplanted hepatocytes by the compound 2‐(2‐nitro‐4‐trifluoro‐methylbenzoyl)‐1,3‐cyclohexanedione (NTBC) to regulate liver injury.^24^, ^25^ FRG mice were crossed with FVIII‐deficient haemophilia A mice to generate a quadruple KO *Fah* ^−/−^ *Rag2* ^−/−^ *IL2rg* ^−/−^FVIII^−/−^ mouse line (FRGF8) (Figure S1C). The FRG8 mice exhibited prolonged activated partial thromboplastin clotting time (aPTT), and the bleeding test led to the death of all mice (Figure S1D).

**FIGURE 3:**
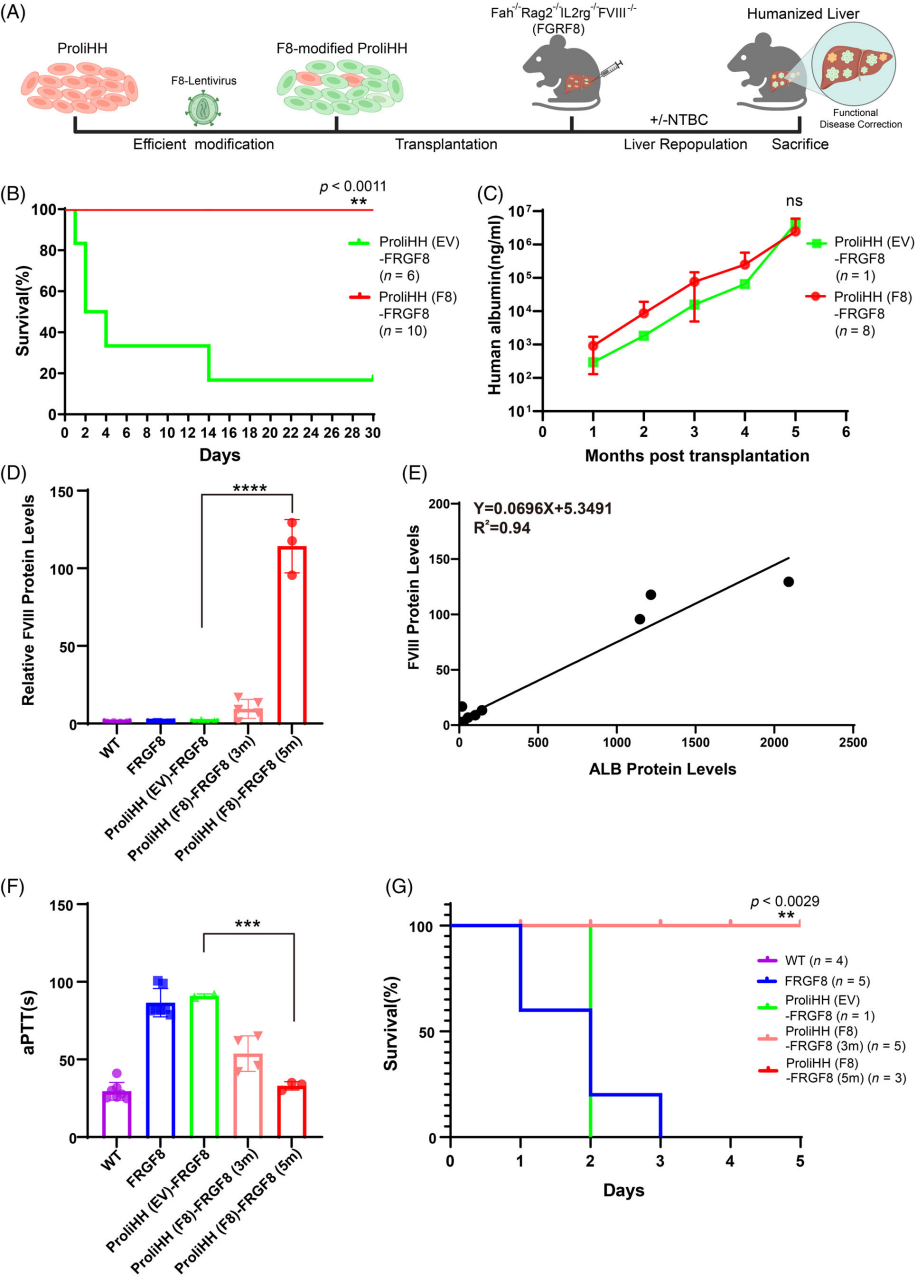
Transplantation of F8‐modified proliferating human hepatocytes (ProliHHs) have a functional rescue in haemophilia A mice. (A) Schematic of F8‐modified ProliHHs transplantation for mice model. ProliHHs were cultured in hepatocyte medium for expansion. cultured ProliHHs were infected to lentivirus (MOI = 1) for F8 expression. F8‐modified ProliHHs were transplanted into FRGF8 mice through spleen transplantation. The expansion of human hepatocytes in vivo was promoted by repeatedly adding withdrawn NTBC in transplanted mice. (B) Kaplan–Meier survival curve of FRGF8 mice with transplantation of 8E5 EV‐modified ProliHHs [ProliHH(EV)‐FRGF8] or F8‐modified ProliHHs [ProliHH(F8)‐FRGF8]. (C) The dynamic process of human ALB levels was determined by ELISA in the survived ProliHH (EV)‐FRGF8 and survived ProliHH (F8)‐FRGF8 mice during 5 months after transplantation. (D) The human FVIII levels was determined by ELISA in wild‐type mice (WT), FRGF8 mice, the survived ProliHH (EV)‐FRGF8 mice at 5 months and survived ProliHH(F8)‐FRGF8 mice at 3 and 5 months after transplantation. (E) Construction of linear regression equation according to each mouse's secreted ALB and FVIII proteins. A linear equation was obtained by linear regression analysis. (F) The plasma human FVIII protein activity was measured by aPTTs. The measurements were performed at 3 and 5 months after transplantation. (G) Measurement of the survival rate within 72 h after the tail clip in mice was performed at 3 and 5 months after transplantation. The data are shown as the mean ± SD. **p < 0.01; ***p < 0.001; log‐rank test for (A) and (G), Student's *t* test for (C), (D) and (F).

With the established animal model, we then transplanted F8‐ProliHHs into FRGF8 mice to examine their therapeutic efficacy (Figure 3A). EV‐ProliHHs were used as the negative control. The results showed that five of the six FRGF8 mice transplanted with EV‐ProliHHs died of surgical bleeding caused by intrasplenic cell transplantation. However, all the mice treated with F8‐ProliHHs transplantation survived, implying that an immediate, effective therapeutic effect had been achieved after F8‐ProliHH transplantation (Figure 3B). We found that the repopulation of genetically modified ProliHHs in mouse liver was promoted by cyclical NTBC withdrawal and restoration. The levels of human albumin in the plasma were gradually increased after transplantation of gene‐modified ProliHHs, reaching a level of 4 mg/mL after 5 months (Figure 3C). This indicated that gene‐modified ProliHHs efficiently engrafted and repopulated mouse livers after transplantation. Moreover, the concentration of secreted human FVIII was improved steadily in mice transplanted with F8‐ProliHHs and was close to the normal FVIII concentrations in the human plasma after 5 months (Figure 3D). By contrast, the human FVIII protein was not detected in the EV‐ProliHH‐transplanted group.

Next, we analysed the relationship between human albumin (ALB) and FVIII concentration. The plot (ALB against FVIII; Figure 3E) revealed a robust positive correlation between the two secretory proteins as analysed by the linear regression model (*R* = 0.94). These results indicated that FVIII concentrations were dependent on the total number of human hepatocytes in F8‐ProliHH mice. Finally, we collected plasma to measure the functional activity of FVIII protein using an aPTT assay.^26^ The aPTTs of the 3‐ and 5‐month‐old F8‐ProliHH groups were reduced to 53 ± 12 s (*n* = 4) and 35 ± 2 s (*n* = 3), respectively, which were significantly shorter than the corresponding values in the five‐month‐old EV‐ProliHH (91 ± 1.800 s, *n* = 2) and untreated group (81.36 ± 19 s, *n* = 8) and were similar to that of the WT group (25.30 ± 6.2 s, *n* = 7) (Figure 3F). After treatment, a tail‐clip challenge was performed to confirm the effectiveness of this therapeutic strategy. Similar to the results observed in the WT group (*n* = 6), all mice transplanted with F8‐ProliHHs survived (*n* = 6), whereas all mice in the untreated (5 out of 5) and EV‐ProliHHs (*n* = 1; because only 1 mouse treated with EV‐ProliHHs transplantation survived after the operation of cell transplantation) died within 72 h after the tail clip challenge (Figure 3G). These results demonstrated that F8‐medicated ProliHHs could effectively restore the expression and activity of the FVIII protein with persistence and significantly correct coagulation function in adult mice with haemophilia A.

### 3.4 Maturation of gene‐modified ProliHHs after repopulation in vivo

**FIGURE 4:**
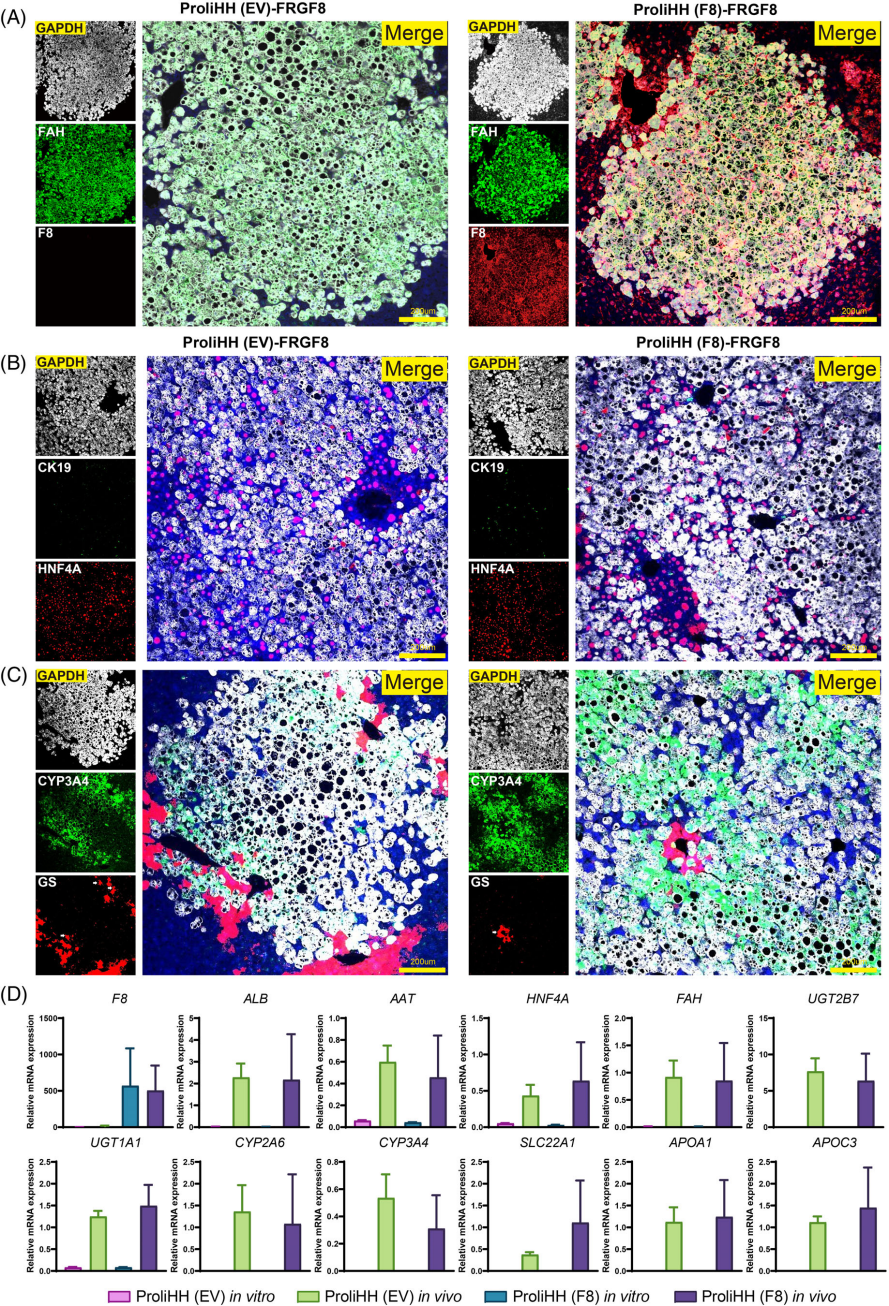
Maturation of gene‐modified proliferating human hepatocytes (ProliHHs) after repopulation in vivo. (A and B) The maturation of repopulated ProliHHs was analysed by co‐immunofluorescent staining for F8, Fah, HNF4a and CK19. (C) The liver zonation was analysed by co‐immunofluorescent staining for Fah, CYP3A4, and GS. Arrows depict human hepatocytes positive for hepatic markers GS and Fah. (D) The Comparison of gene expression of mature hepatic markers, such as phase I, phase II enzymes, and transporters genes in PHHs, EV‐ProliHHs, repopulated EV‐ProliHHs in mice liver, and F8‐ProliHHs, repopulated F8‐ProliHHs in mice liver. RNA was extracted from repopulated livers. Human‐specific primers were used in qPCR.

Gene‐modified ProliHHs are bi‐phenotypic cells, they retained some of the features of mature hepatocytes and expressed liver progenitor‐associated markers. That is, they do not exhibit fully mature hepatic functions.^19^ Here, we further examined whether gene‐modified cells could transform into mature hepatocytes after transplantation. Firstly, F8‐Protein was detected by immunofluorescence (IF) staining in F8‐ProliHHs mice (Figure 4A).

Immunofluorescent staining in the liver tissue of F8‐ProliHHs mice showed that repopulated F8‐ProliHHs expressed the mature hepatocyte markers FAH and HNF4A and inhibited the expression of CK19, indicating the loss of characteristics of liver progenitor cells (Figure 4A,B). Moreover, pericentral zonation markers CYP3A4 and GS were detected in repopulated F8‐ProliHHs in the pericentral area, indicating that F8‐ProliHHs underwent maturation and establishment of a zonation‐specific expression profile (Figure 4C). To evaluate the maturity of repopulated F8‐ProliHH in the treated mice, we checked the hepatic gene expression of repopulated gene‐modified ProliHHs by qPCR using human‐specific primers. The repopulated gene‐modified ProliHHs showed significantly higher expression of mature hepatic genes, including phase I and II enzymes and transporters, at levels comparable to PHHs after transplantation (Figure 4D). Together, these data suggested that gene‐modified ProliHHs underwent maturation with human‐specific metabolism in vivo.

### 3.5 Integration profile of F8‐modified ProliHHs

In our previous study, no tumours were found in ProliHH‐transplanted FRG mice after transplantation.^19^ Nevertheless, whether there are potential genotoxic effects associated with the lentivirus‐modified ProliHHs remains unclear. Therefore, the livers of F8‐modified ProliHH‐transplanted FRGF8 mice were examined 5 months after transplantation. We did not observe macroscopic liver tumours in any of the treated mice.

**FIGURE 5:**
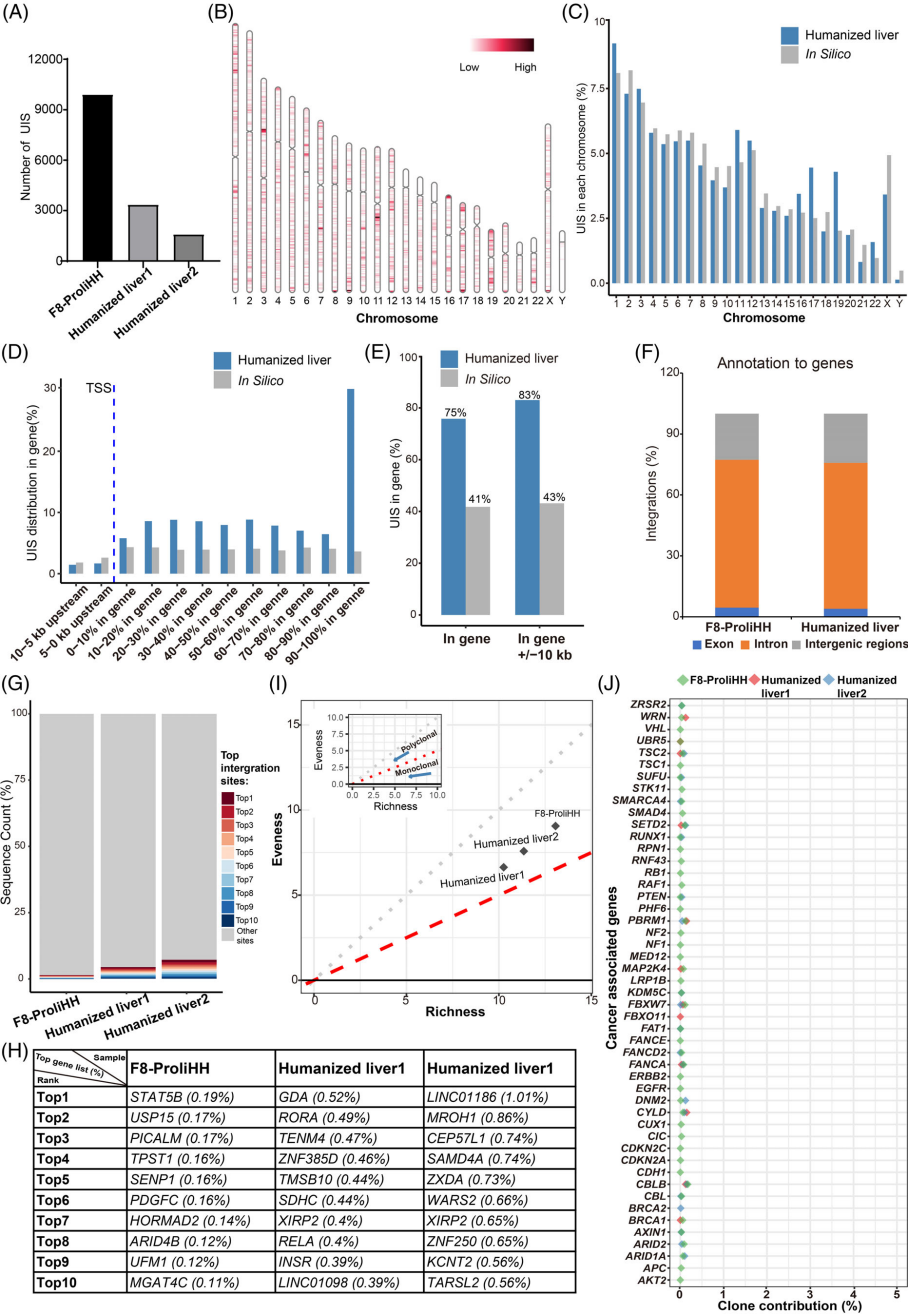
Integration profile of F8‐modified proliferating human hepatocytes (ProliHHs). (A) Integration numbers were identified in libraries obtained from F8‐ProliHHs and F8‐ProliHHs repopulated mice livers (humanized livers) using two long terminal repeat primers. (B) Integrations were mapped to the human genome and distribution of lentivirus integration sites in the human chromosomes. Chr, chromosome. (C) Integration site proportions were counted in each human chromosome. Bars in grey represent percentages corresponding to a random distribution of unique integration sites (UISs) in silico, while blue bars represent actual proportions of UISs determined in the two humanized livers. (D) Distribution of the UISs determined in genes and genes ±10 kb. TSS, transcription start site. (E) 75% UISs were observed in gene regions, while 83% UISs were observed in gene ±10 kb regions. No clustering around TSS regions was observed. Bars in grey represent percentages corresponding to a random distribution of UISs in silico, while blue bars represent actual proportions of UISs determined in the two humanized livers. (F) Percentage of integrations mapped to exons, introns, and intergenic regions. (G) The proportions of the 10 most represented UISs in F8‐ProliHHs and F8‐ProliHHs repopulated mice livers (humanized livers). (H) The gene list and proportions of the top 10 UISs in F8‐ProliHHs and F8‐ProliHHs repopulated mice livers (humanized livers). (I) Polyclonal‐monoclonal distance (PMD) clonal framework. PMD analysis is conducted from two dimensions: evenness and richness for the clonal diversity of samples. (J) Clone proportions of UISs at 100 kb upstream and downstream around TSS regions of Cancer associate gene.

To investigate the risk of oncogenesis, we evaluated the clonal nature of engrafted F8‐modified ProliHHs in transplanted FRGF8 mice. We sequenced the pre‐transplanted cells (F8‐modified ProliHHs) and two samples of the post‐transplanted humanized liver using ligation target amplification PCR (LTA‐PCR) to collect the genome‐wide lentiviral integration profile for clone analysis.^27^, ^28^, ^29^ After mapping to specific locations in the genome using the ISoverTime tool, we identified 9902, 3355 and 1587 unique integration sites (UISs) in F8‐modified ProliHHs and two liver samples of engrafted F8‐modified ProliHHs, respectively (Figure 5A). We then performed a global analysis of integration site distributions. The results of the gene mapping analysis showed that integration events were widely distributed throughout the human chromosomes and were not exactly consistent with a random distribution of UISs using in silico data (Figure 5B,C). To identify integration site preferences within the gene, integrations were mapped to exons, introns, and intergenic regions. We found that 75.8% of the UISs occurred within genes in which integrations were mainly located in introns (~72%) (Figure 5D–F). These preferential lentiviral integrations within transcriptional units were inconsistent with the random lentivirus integration of computer simulations.

To determine whether some F8‐modified ProliHH clones have a selective growth advantage in vivo, we analysed the proportion and insertion position of the top10 enriched F8‐modified ProliHH clones, and no clear ProliHH clones were found in any samples (Reference Standards <30%) (Figure 5G,H). Furthermore, clonal diversity analysis also showed that clone diversity was retained in transplanted populations, indicating that liver transplanted F8‐modified ProliHHs maintained a diverse state (Figure 5I). Importantly, integrations in selected cancer‐related genes did not promote clone growth (Reference Standards <5%), and no related dominating clones were found in humanized liver analysed by the proportion of these ProliHH clones (Figure 5J). Overall, these results did not show evidence of genotoxicity and demonstrated the safety of F8‐modified ProliHH transplantation in the mouse model.

## 4 DISCUSSION

Owing to its broad therapeutic range, haemophilia A remains an ideal target for alternative therapies, such as gene and cell therapy. AAV‐based gene therapies targeting hepatocytes have become increasingly successful approaches to treating haemophilia in recent years. In addition, clinical and preclinical studies have already demonstrated the safety and short‐term success of hepatocyte transplantation in patients with liver metabolic disorders.^13^, ^14^ Therefore, F8‐modified hepatocyte transplantation may be a promising strategy to treat haemophilia A. In the present study, we found that ProliHHs were more susceptible to lentiviral infection than PHHs. ProliHHs effectively secreted F8 proteins and maintained their bi‐phenotypic status after F8‐lentivirus modification. Importantly, F8‐modified ProliHHs showed a remarkable capability to repopulate and significantly corrected coagulation function in adult mice with haemophilia A after in vivo transplantation. As a proof of concept, our study provided a new approach for the treatment of haemophilia A.

A major goal of cell therapy is to replace deficient functions with healthy cell transplantation. Liver sinusoidal endothelial cells are known to be the main producers of FVIII,^15^, ^16^, ^17^ and several studies have shown that endothelial cells repopulated the liver endothelium and corrected the bleeding phenotype of haemophilia A mice.^30^, ^31^ Unfortunately, liver sinusoidal endothelial cell transplantation has not been successfully achieved in clinical treatments, owing to the uncertainty regarding safety and effectiveness of cell transplantation. In fact, clinically feasible cell transplantation therapies may be applied to haemophilia treatment, such as haematopoietic stem cell and hepatocyte transplantation.^10^ Currently, a clinical study is evaluating the effectiveness of transplanting autologous CD34+ haematopoietic stem cells transduced with the CD68‐ET3 LV for treating severe haemophilia A (NCT04418414). To date, over 100 cases of clinical hepatocyte transplantation have been reported, and definite clinical improvements have been documented in patients transplanted with hepatocytes.^32^ Therefore, F8‐modified hepatocyte transplantation represents a therapeutically attractive and practical strategy for treating haemophilia A.

LVs provide an efficient means for the modification of multiple primary cells, especially in research and clinical gene therapy applications. However, safety remains one of the most critical concerns, because of semi‐random integration and stimulation of immune responses. A previous clinical trial demonstrated that viral integration patterns near proto‐oncogenes could increase the risk of leukemogenesis.^33^ Therefore, several improvements have been made to LVs to improve the safety and regulate transgene expression. The latest clinical trial results showed that newly developed vectors strongly reduced the risk of viral‐mediated carcinogenesis, as no patients with leukaemia were reported in gene therapy trials involving genetically modified HSC or T cells.^34^, ^35^, ^36^ With advances in genome sequencing techniques, genome‐wide surveys of viral integration sites, and monitoring of in vivo expansion and the proportion of transplanted cells with a specific integration site have become possible. Hirsch et al. analysed the integration profile of cultivated MLV‐RV‐transduced keratinocytes, and reported no immortalization events related to specific lentiviral integrations.^37^ Here, through the analysis of the integration profile of genetically modified hepatocytes, we also found that no oncogenic clones with growth advantages were observed in any of the clones of gene‐modified hepatocytes. Moreover, the treated mice did not manifest tumour development or other related adverse events, indicating a low risk of insertional oncogenesis in genetically modified ProliHHs, similar to the findings of transgenic epidermis and HSCs transplantation.

According to our previous HM culture system, ProliHHs are expandable human hepatocytes with bi‐phenotypic status. Genetically modified ProliHHs can acquire new abilities or correct defective functions without changing the biological characteristics of cells via lentivirus infection. In this study, we demonstrated that the F8‐modified ProliHHs obtained a new ability to secrete F8 proteins, and transplantation of the cells could effectively correct the bleeding phenotype in mice with haemophilia A. Similarly, we hypothesize that autologous ProliHHs derived from patients with genetic liver diseases can be corrected or gene‐edited by lentivirus to treat these diseases. This therapeutic strategy of using autologous gene‐corrected ProliHHs could compensate for the poor long‐term effectiveness of allogeneic hepatocyte therapy caused by immune rejection. However, we realize that implementing this new treatment strategy will be challenging. It needs to meet the requirements of massive expansion of patient hepatocytes, effective gene correction, and improvement of hepatocyte transplantation technology.

In conclusion, our study provides a new proof of concept that ex vivo gene therapy of human ProliHHs by lentiviral infection is feasible. Furthermore, we evaluated the safety and efficacy of the modified ProliHHs using animal models. In addition, these modified human hepatocytes may be used for extensive studies of cell therapy in vivo and liver disease modelling in vitro.

## AUTHOR CONTRIBUTIONS

Kun Zhang designed and performed most of the experiments. Ning Wu and Kun Zhang analysed the data of integration sites. Jing Cen performed the hepatocyte transplantation. Jie Li assisted cell culture and immunofluorescence staining. Zhen Wang performed the analysis of hepatic genes in vivo. Kun Zhang analysed the data and wrote the manuscript. Lijian Hui supervised and coordinated the project.

## FUNDING INFORMATION

This project was supported by ‘Strategic Priority Research Program’ of the Chinese Academy of Sciences (CAS, XDA16020201), the National Science Foundation of China (NSFC) (32001070), the Special Research Assistant Project of the Chinese Academy of Sciences (E019710101) and China Postdoctoral Science Foundation (2019M660101, 2019TQ0339). The integration profiles were analysed in Waker Bioscience. The graphical abstract was adapted from BioRender.com (2023).

## CONFLICT OF INTEREST STATEMENT

The authors declare no competing interests.

## Supporting information


**Figure S1.** Preparation of lentivirus plasmid and animal model. (A) F8 and F9 gene expression were analysed by ProliHHs RNA‐seq data. primary human hepatocytes (PHHs), Proliferating human hepatocytes (ProliHHs) and transplanted ProliHHs in FRG mice (ProliHHs in vivo), the data source is from GSE112866.^19^ (B) Schematic of F8‐lentivirus vectors carrying a EF1a promoter, a codon optimized DNA encoding a B domain‐deleted (BDD) human FVIII protein, and a reporter GFP. (C) Schematic of FRGF8 mice line crossed from FRG mice and FVIII deficient haemophilia A mice. (D) The plasma FVIII protein activity was measured by aPTTs in wild‐type (WT) mice, FRG and FRGF8.
**Table S1.** Primer sequences for qPCR, Relative to Methods.

## Data Availability

The authors confirm that the data supporting the findings of this study are available within the article and its supplementary materials.
