# Supplementary material for: Ex vivo factor VIII‐modified proliferating human hepatocytes therapy for haemophilia A
Source: Cell Prolif. 2023 May 17;56(5):e13467. doi: 10.1111/cpr.13467 (PMC10212703; doi:10.1111/cpr.13467)
Supplement: Supplementary file 1 — Figure S1. Preparation of lentivirus plasmid and animal model. (A) F8 and F9 gene expression were analysed by ProliHHs RNA‐seq data. primary human hepatocytes (PHHs), Proliferating human hepatocytes (ProliHHs) and transplanted ProliHHs in FRG mice (ProliHHs in vivo), the data source is from GSE112866. 19 (B) Schematic of F8‐lentivirus vectors carrying a EF1a promoter, a codon optimized DNA encoding a B domain‐deleted (BDD) human FVIII protein, and a reporter GFP. (C) Schematic of FRGF8 mice line crossed from FRG mice and FVIII deficient haemophilia A mice. (D) The plasma FVIII protein activity was measured by aPTTs in wild‐type (WT) mice, FRG and FRGF8. Table S1. Primer sequences for qPCR, Relative to Methods. [file CPR-56-e13467-s001.docx]

***Ex vivo*** **Lentiviral Factor VIII-modified ProliHHs therapy for Haemophilia A**

Kun Zhang, Ning Wu, Jing Cen, Jie Li, Zhen Wang, Qiang Xia, and Lijian Hui

**
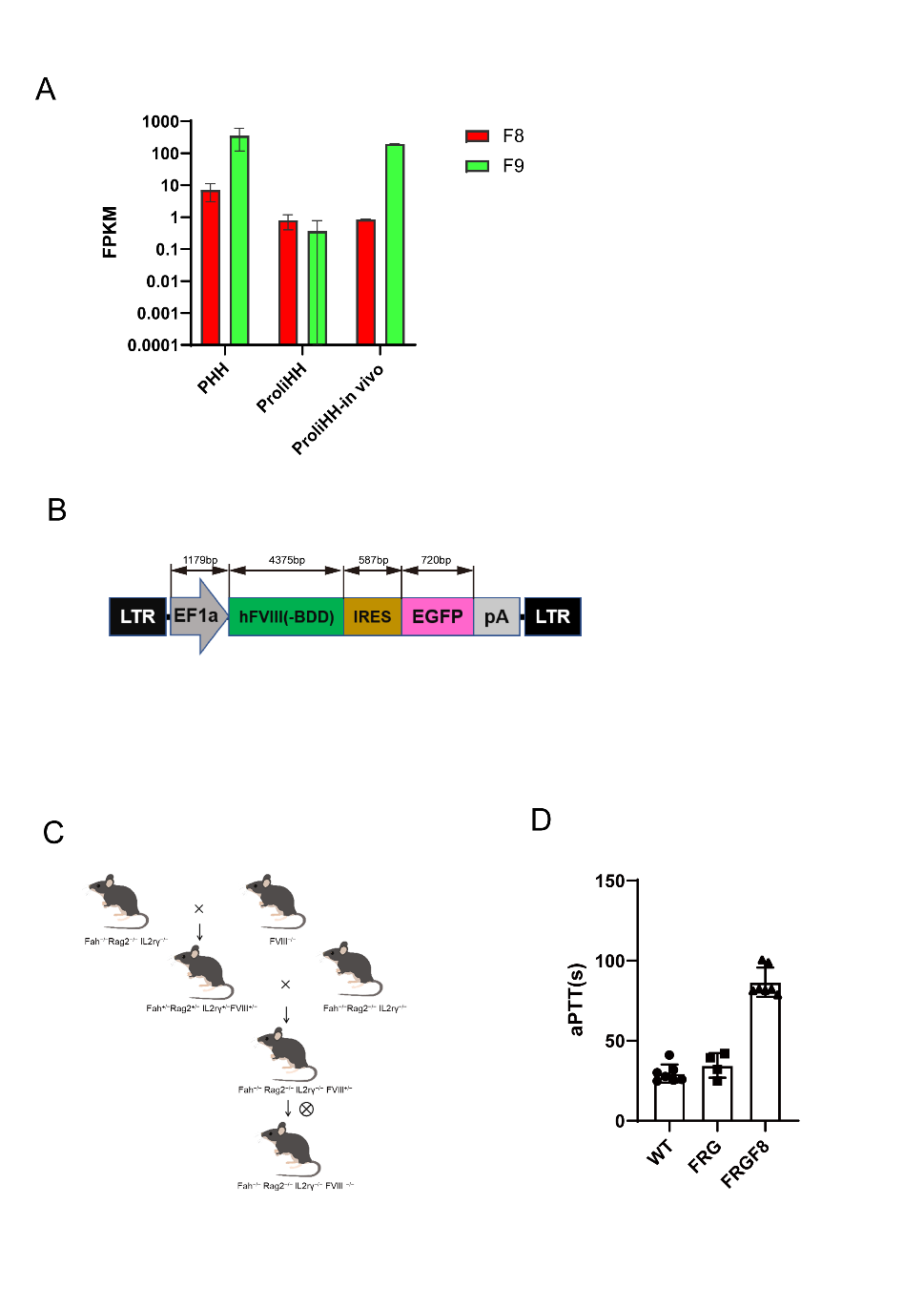
Supplementary figure and legends**

**Supplementary figure 1: Preparation of lentivirus plasmid and animal model**

(A) F8 and F9 gene expression were analyzed by ProliHHs RNA-seq data. primary human hepatocytes (PHHs), Proliferating human hepatocytes (ProliHHs) and transplanted ProliHHs in FRG mice (ProliHHs in vivo)，the data source is from GSE112866. ^19^ (B)Schematic of F8-lentivirus vectors carrying a EF1a promoter, a codon optimized DNA encoding a B domain deleted (BDD) human FVIII protein, and a reporter GFP. (C)Schematic of FRGF8 mice line crossed from FRG mice and FVIII deficient hemophilia A mice. (D) The plasma FVIII protein activity was measured by aPTTs in wild type mice (WT), FRG and FRGF8.

**Supplemental Table 1. Primer sequences for qPCR, Relative to Methods.**

| Gene | Forward(5'-3') | Reverse(5'-3') |
| --- | --- | --- |
| ALB | GCCTTTGCTCAGTATCTT | AGGTTTGGGTTGTCATCT |
| AAT | TATGATGAAGCGTTTAGGC | CAGTAATGGACAGTTTGGGT |
| TTR | TGGGAGCCATTTGCCTCTG | AGCCGTGGTGGAATAGGAGTA |
| CYP1A2 | CTTCGCTACCTGCCTAACCC | GACTGTGTCAAATCCTGCTCC |
| CYP2A6 | CAGCACTTCCTGAATGAG | AGGTGACTGGGAGGACTTGAGGC |
| CYP2B6 | GCACTCCTCACAGGACTCTTG | CCCAGGTGTACCGTGAAGAC |
| CYP2D6 | TGAAGGATGAGGCCGTCTGGGAGA | CAGTGGGCACCGAGAAGCTGAAGT |
| CYP3A4 | TTCAGCAAGAAGAACAAGGACAA | GGTTGAAGAAGTCCTCCTAAGC |
| F8 | ATTCTGGGGTGCCACAACTC | CTGGTGGGTTTTGAGAGAAGC |
| CD133 | TACCAAGGACAAGGCGTTCAC | CAGTCGTGGTTTGGCGTTGTA |
| APOA1 | CTTTGGCTTCAGCTTCAAGG | ATCCTGAGGGAATGTCGATG |
| HNF4A | GCGTCTCGCCAGATTGAGG | GAGGCACCGTAGTGTTTGC |
| FAH | CCTACGGCGTCTTCTCGAC | CTGCAAGAACACTCTCGCCT |
| AFP | ACTGAATCCAGAACACTGCA | TGCAGTCAATGCATCTTTCA |
| GAPDH | CCACCTTTGACGCTGGG | CATACCAGGAAATGAGCTTGACA |
| EPCAM | AGGAGATGGGTGAGATGC | GATTGGTAAAGCCAGTTTC |
| LGR5 | TCCACTTTGCCATCCCTAA | GGTCGTCCATACTGCTGTTG |
| CFTR | TGAAACTGACTCGGAAGG | CAGAATGAGATGGTGGTG |
| CK19 | TCCGAACCAAGTTTGAGACG | CCCTCAGCGTACTGATTTCCT |
| SOX9 | GACTACACCGACCACCAGAACTCC | GTCTGCGGGATGGAAGGGA |
| UGT1A1 | CATGCTGGGAAGATACTGTTGAT | GCCCGAGACTAACAAAAGACTCT |
| GSTA1 | CTGCCCGTATGTCCACCTG | AGCTCCTCGACGTAGTAGAGA |
| APOC3 | GAAGCACGCCACCAAGAC | CAGGGTCCAAATCCCAGAA |
| UGT2B7 | AAGGTGCTGGTGTGGGCAG | AGCGGATGAGTTGTTGGGA |
| CYP2E1 | ATGTCTGCCCTCGGAGTCA | CGATGATGGGAAGCGGGAAA |
| CK7 | GAAGCATGGGGACGACCT | CACGAGCATCCTTGAGCG |
